# Peptide-templated noble metal catalysts: syntheses and applications

**DOI:** 10.1039/c7sc00069c

**Published:** 2017-02-14

**Authors:** Wei Wang, Caleb F. Anderson, Zongyuan Wang, Wei Wu, Honggang Cui, Chang-Jun Liu

**Affiliations:** a Tianjin Co-Innovation Center of Chemical Science & Engineering , School of Chemical Engineering and Technology , Tianjin University , Tianjin 300072 , China . Email: ughg_cjl@yahoo.com; b International Joint Research Centre for Catalytic Technology , Key Laboratory of Chemical Engineering Process & Technology for High-Efficiency Conversion , School of Chemistry and Material Science , Heilongjiang University , Harbin 150080 , China; c Department of Chemical and Biomolecular Engineering , Institute for NanoBioTechnology , Johns Hopkins University , Baltimore , MD 21218 , USA

## Abstract

Noble metal catalysts have been widely used in many applications because of their high activity and selectivity. However, a controllable preparation of noble metal catalysts still remains as a significant challenge. To overcome this challenge, peptide templates can play a critical role in the controllable syntheses of catalysts owing to their flexible binding with specific metallic surfaces and self-assembly characteristics. By employing peptide templates, the size, shape, facet, structure, and composition of obtained catalysts can all be specifically controlled under the mild synthesis conditions. In addition, catalysts with spherical, nanofiber, and nanofilm structures can all be produced by associating with the self-assembly characteristics of peptide templates. Furthermore, the peptide-templated noble metal catalysts also reveal significantly enhanced catalytic behaviours compared with conventional catalysts because the electron conductivity, metal dispersion, and reactive site exposure can all be improved. In this review, we summarize the research progresses in the syntheses of peptide-templated noble metal catalysts. The applications of the peptide-templated catalysts in organic reactions, photocatalysis, and electrocatalysis are discussed, and the relationship between structure and activity of these catalysts are addressed. Future opportunities, including new catalytic materials designed by using biological principles, are indicated to achieve selective, eco-friendly, and energy neutral synthesis approaches.

## Abstract 2

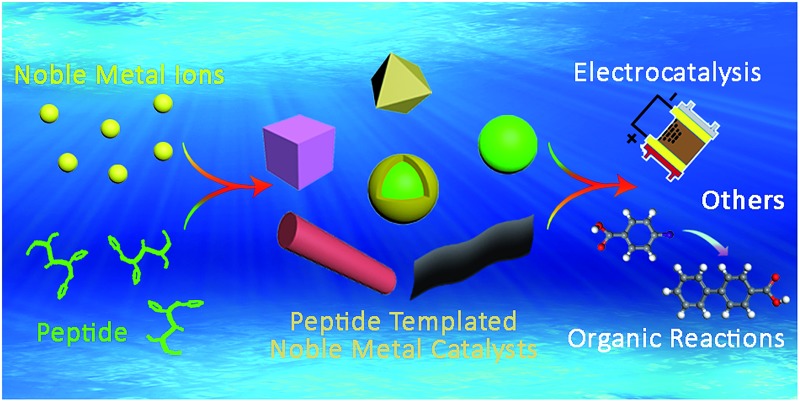
Peptide templates can play a critical role in the controllable syntheses of catalysts owing to their flexible binding with specific metallic surfaces and self-assembly characteristics.

## 1 Introduction

In recent years, noble metal catalysts with at least one dimension within the nanoscale have possessed outstanding catalytic behaviours in many applications such as organic reactions, photocatalysis, electrocatalysis, and many others.^1–5^ Compared with their bulk counterparts, the higher catalytic activity of nanostructured noble metal catalysts generally originates from the larger surface-to-volume ratio. With a larger surface/volume ratio, more metallic atoms are exposed as efficient reactive sites, and thus the activity of the catalysts is substantially enhanced.^6–10^ Therefore, the fabrication of noble metal catalysts with precise control over multiple properties including size, shape, facet, structure, and composition has long been a central topic in the field of nanomaterial science and application.^11,12^

Nowadays, significant progress has been made in the controllable syntheses of noble metal catalysts, which typically use either of the following complementary strategies: the “top-down” approach or the “bottom-up” approach.^13–15^ By using the “top-down” approach, a bulk noble metal material is reduced down to nanoscale particles. This approach can control size and shape of nanoparticles precisely, but the layer-by-layer or point-by-point procedure takes a relatively long time and has a high energy cost.^13^ In contrast, the “bottom-up” fabrication of noble metal catalysts involves two steps: single atom formation and aggregation of atoms to nanoparticles. The “bottom-up” approach is simple and fast, and the building blocks can be designed to facilitate the assembly of nanoparticles with specific features. For this approach, however, conditions such as high temperature, non-natural protective agents, or continuous vigorous stirring are essential, causing potential environmental concerns and a high energy expenditure.^16^ Therefore, it is still necessary to develop the “bottom-up” approach to achieve mild operation conditions with environmentally-benign capping agents.

**Fig. 1:**
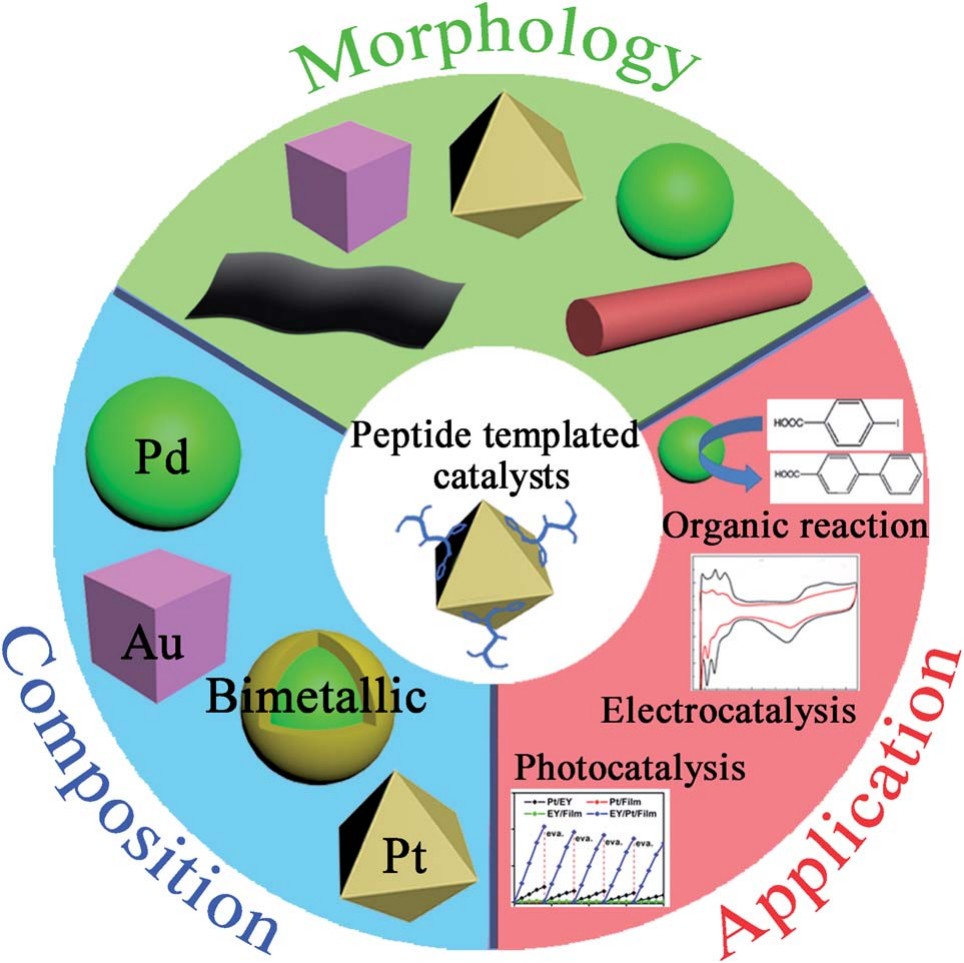
Characteristics of peptide-templated noble metal catalysts, including morphologies, compositions, and applications.

Over the past 15–20 years, many researchers have turned to nature for inspiration to further develop the “bottom-up” approach.^17–21^ Among various biomimetic and bioinspired synthesis studies, peptides have been demonstrated as an effective template to prepare noble metal catalysts with characteristics of strict control and high catalytic performance (Fig. 1).^22–24^ There are many advantages for choosing peptide templates as the capping agent.^16,17^ The first advantage is that the fabrication conditions of peptide-templated synthesis are generally at room temperature, in aqueous solutions, and near neutral pH, which are much milder compared with traditional synthesis techniques. This evident improvement makes catalyst fabrication processes much “greener” because of the lower energy input and avoidance of non-natural solvents. Secondly, the peptide template can specifically control the size, shape, facet, and structure of the obtained catalysts due to the different adsorbed motifs and interactions between the peptide and the specific metallic surface. The third advantage is the large diversity of natural and artificial peptide sequences that provide numerous options to produce the required catalysts. If necessary, researchers may even synthesize a desired peptide sequence based on the known effect of different amino acids on a specific metallic surface. Finally, owing to the high electron conductivity of peptide templates, the catalytic activity of prepared noble metal catalysts is significantly enhanced in photocatalysis and electrocatalysis. Due to these advantages, peptide-templated synthesis has been developed as a novel and important approach to produce noble metal catalysts.

In this review, we attempt to summarize the recent advances in the peptide-templated synthesis of noble metal catalysts, including the interactions and binding mechanisms between peptides and metallic surfaces. The applications of prepared catalysts for organic reactions, photocatalysis, and electrocatalysis are also discussed. The summarized studies reveal the great potential for adapting peptide template synthesis toward efficient noble metal catalysts for chemical syntheses, sustainable energy applications, and more.

## 2 Synthesis of peptide-templated noble metal catalysts

Recently, studies on biomimetic syntheses of peptide-templated noble metal nanoparticles have demonstrated that peptides can effectively and specifically control the properties of nanoparticles for catalytic applications.^25–27^ Such functions of peptide templates originate from their flexible and strong binding with specific metallic surfaces, which has been proven by theoretical and experimental studies.^28–30^ According to these findings, a novel and effective route has been established to synthesize noble metal catalysts more naturally.

### 2.1 Peptide-templated noble metal nanoparticles

To date, chemical reduction is still the most widely used method to synthesize peptide-templated noble metal catalysts.^31–34^ Sodium borohydride (NaBH_4_) or ascorbic acid is typically employed as the reducing agent, while the peptide plays the role of capping agent. In some cases, the peptide itself can be the reducing agent. The general operation steps are as follows. First, a pre-mixed solution containing the metal ion precursor and peptide in appropriate proportions is prepared. Then, a small quantity of reducing agent is added into this solution for metal ion reduction. Generally, no further steps, with the exception of stirring for a few hours, are needed to obtain nanoparticles with the targeted morphology. Additionally, the whole synthesis process is under mild aqueous conditions and simple to operate. Simultaneously, various nanoparticles with controlled morphologies could be produced by simple alterations to the fabrication conditions, such as peptide sequence, metal/peptide ratio, pH value, and reaction time.

**Fig. 2:**
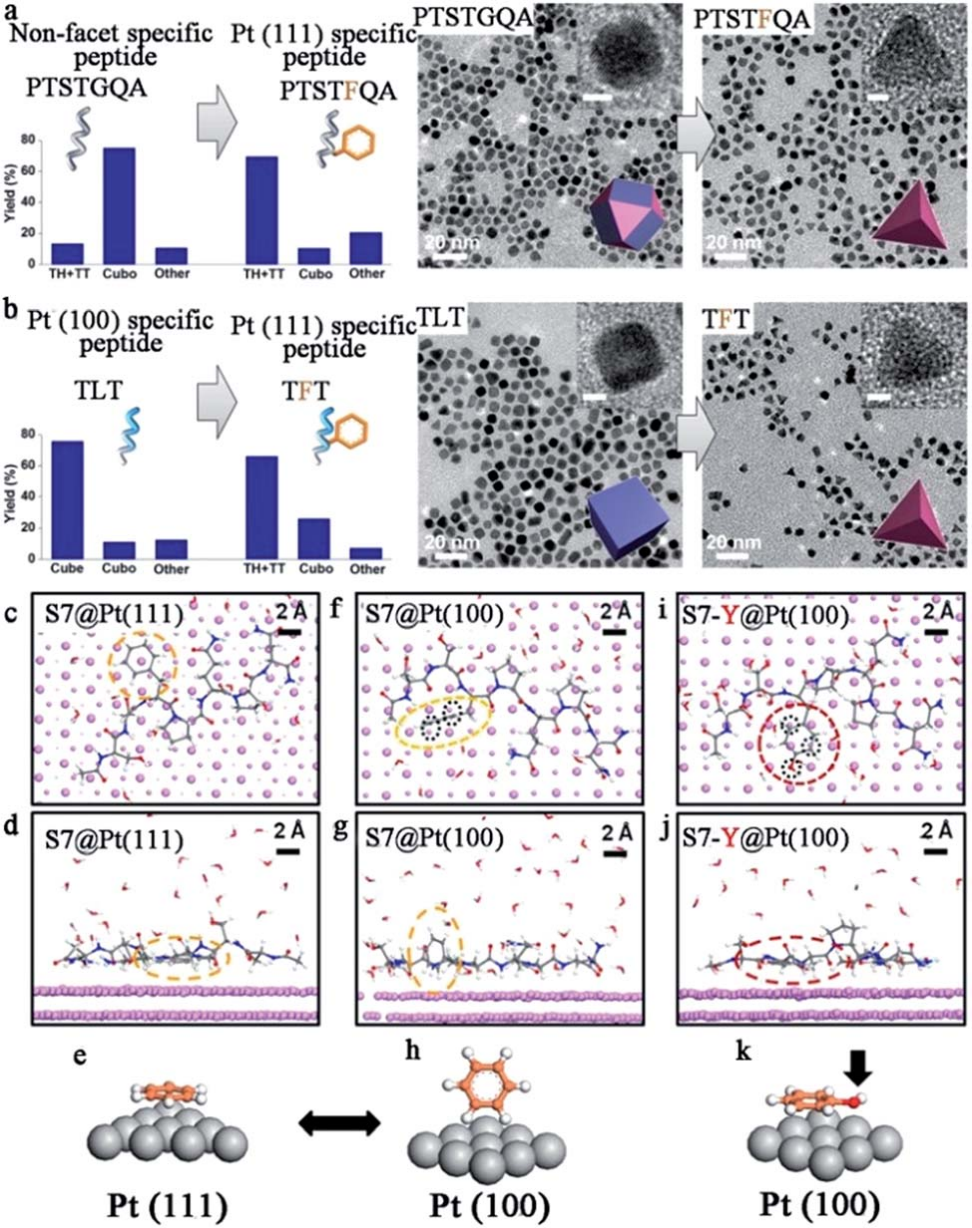
Biomimetic design and the origin of Pt (111) specificity with peptide templates. (a) Demonstration of turning a non-facet specific Pt binding peptide (PTSTGQA) into a Pt (111)-specific peptide (PTSTFQA) through inclusion of the phenyl ring; (b) demonstration of switching a Pt (100)-specific peptide (TLT) into a Pt (111)-specific peptide (TFT) by applying the phenyl ring. The yields of Pt NPs with different shapes are shown on the left side; (c–e, f–h) binding configurations of S7 peptide on Pt (111) and (100) surfaces (top view and side view). Configurations of the phenyl ring are highlighted in dashed yellow circles and also schematically illustrated in (e) and (h). (i–k) Binding configurations of S7-Y peptide on Pt (100) surface (top view and side view). Configurations of the phenol ring are highlighted in dashed red circles and schematically illustrated in (k). Black circles in (f) and (i) indicate the differences in epitaxial contacts between phenyl and phenol rings on Pt (100) surfaces, which account for the difference in their binding energy, Ea (100). Most water molecules are omitted for visual clarity. This figure is reproduced with permission from ref. 33, copyright American Chemistry Society.

By using peptide-templated synthesis, Huang and her co-workers fabricated a series of Pt catalysts with high electrocatalytic activity.^31–35^ During the fabrication process, chloroplatinic acid (H_2_Pt(iv)Cl_6_) was used as the precursor, while ascorbic acid and sodium borohydride (NaBH_4_) were used as mild and strong reducing agents, respectively. They employed Pt (100) binding peptide T7 (Ac-TLTTLTN-CONH_2_) to produce cubes enclosed by six (100) facets, and Pt (111) binding peptide S7 (Ac-SSFPQPN-CONH_2_) to produce tetrahedrons enclosed by four (111) facets.^32^ After the rapid, room temperature fabrication process, the size of the prepared Pt nanoparticles was only 3–7 nm. Furthermore, the shape of the prepared Pt nanoparticles was controlled perfectly, since the T7 peptide (Pt (100) binding) and S7 peptide (Pt (111) binding) can effectively limit the growth along (100) and (111) facets and consequently facilitate the formation of (100) and (111) facet enclosed nanoparticles, respectively.^32^ In order to prove the hypothesis regarding the effect of the peptide templates, they performed additional theoretical and experimental studies to understand the molecular mechanisms of specific recognition of different shape Pt nanoparticles by peptide templates.^33,34^ As shown in Fig. 2, they found that phenylalanine is the dominant motif to differentiate the Pt (111) facets.^33^ When adsorbing onto the Pt (111) surface, the phenyl ring adopts a flat configuration parallel to the surface due to a much lower adsorption energy, and in doing so prevents the growth of Pt nanoparticles along the (111) facet. However, for the (100) facet, the phenyl ring would exhibit a “stand-up” configuration that could not stop the growth along the (100) facet. As a result, the Pt nanoparticles adopt a tetrahedron shape enclosed by (111) facets.^33^ In addition, by adding phenylalanine into the peptide sequence, the shape of prepared Pt nanoparticles could be switched effectively.^33^ Besides Pt nanocubes and nanotetrahedrons, Ruan *et al.* also produced Pt nanoparticles with other shapes by using different peptide sequences.^35^ For example, the prepared Pt nanoparticles were mainly twinned multipod morphologies when employing TLHVSSY, THSVSLY and TLHV peptide sequences, and mainly single-crystalline cuboctahedra morphologies when employing SSY, TLGVSSY and TLGV peptide sequences.^35^ Beyond the Pt monometallic catalyst, they also produced PtNi and PtNiCo bi- and tri-metallic catalysts for electrocatalysis, demonstrating that the peptide template can also provide a specific control effect for bimetallic catalyst fabrication.^36^

**Scheme 1:**
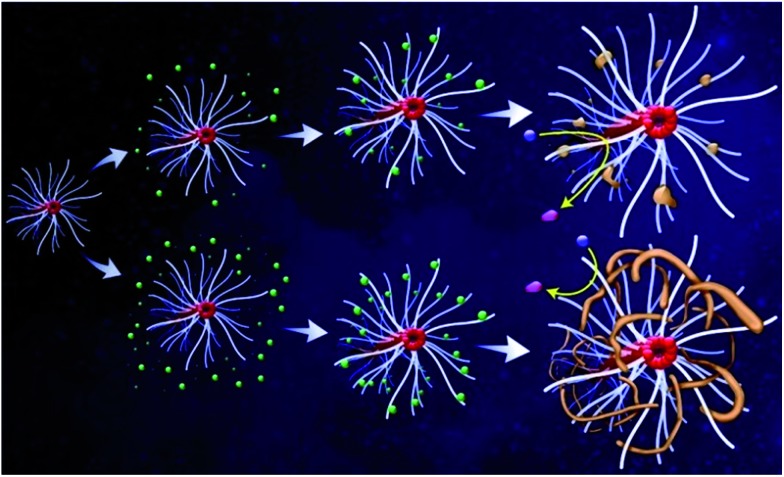
Synthetic scheme of Pd catalyst formed with different peptide template amounts. The top pathway reveals the fabrication of Pd catalysts with low Pd/peptide ratios, while the bottom pathway indicates the preparation of Pd catalysts with high Pd/peptide ratios. In this scheme, the green spheres represent Pd^2+^ ions that interact with the peptide template, which are then reduced to Pd metallic nanoparticles. This scheme is reprinted with permission from ref. 38, copyright American Chemistry Society.

Besides Pt catalysts, Knecht and Naik also studied many ways to fabricate peptide-templated Pd and Au catalysts with the investigation of peptide effects by theoretical and experimental studies.^37–40^ Similarly to Pt catalysts, they proved that the size, shape, and morphology of Pd and Au catalysts can be specifically controlled by different peptide sequences. For making Pd catalysts, Pd4 (TSNAVHPTLRHL) and R5 (SSKKSGSYSGSKGSKRRIL) peptides were used as the templates, with K_2_PdCl_4_ as the precursor and NaBH_4_ as the reducing agent.^37,38^ They found that the formation mechanism of peptide-templated Pd nanoparticles is very similar to that of the Pt catalyst, being directly dependent on the adsorbed motifs of peptides on the Pd surface.^37^ According to their EXAFS results, the coordination numbers (CNs) for Pd–S/Cl are much higher than those for Pd–O/N, demonstrating the high affinity of the peptide template to the Pd surface. Since the cysteine residues of the peptide sequence possess more S and Cl atoms than other residues, the EXAFS results indicate that the peptide is adsorbed onto the Pd surface through the strong interactions between the cysteine residues and metallic sites. The remaining residues of the peptide are then arranged to provide optimal surface binding to minimize the surface energy.^37^ Beyond experimental measurements, they further developed molecular dynamics (MD) simulations by introducing a reverse Monte Carlo (RMC) simulation. As the RMC simulation is an ideal technique for building a configuration model of highly disordered materials based on experimental data, the modified MD simulation with the RMC model can relax atomic positions and achieve stable morphologies to provide more accurate theoretical results.^37^ In addition, Knecht *et al.* also studied the influence of Pd/peptide ratios during Pd catalyst fabrication processes.^38^ They found that the morphology of Pd catalysts changes from spherical, linear/ribbon-like to nanoparticle network (NPN) structures as the Pd/peptide ratios increase from 60 to 120. Scheme 1 shows the formation mechanisms of different Pd structures with various peptide additions. It can be seen that when the Pd/peptide ratio is lower, the Pd ions are more widely separated and, after reduction to Pd nanoparticles, the greater particle distances can effectively prevent aggregation. However, for higher Pd/peptide ratios, the particle distances are evidently shorter such that nanoparticles will specifically aggregate into a linear structure along the branches of the peptide template. With the Pd/peptide ratio continuously increasing, more linear aggregation occurs to form the network morphology and to finally generate the NPN structure.^38^ From this study, it can be concluded that not only 0-D nanostructures, but also 1-D and 2-D nanostructures of noble metal catalysts can be formed just by increasing the metal/peptide ratios. According to the mechanism discussed above, the template effects of peptides are very similar to those of conventional protective agents, which control the characteristics of Pd catalysts by adsorbing on the surface of Pd NPs. However, in addition to covering all metal surfaces as conventional protective agents do, the peptide-templated Pd catalysts could potentially expose more reactive metal sites. Once the concentration of the peptide template is increased above a threshold value, the peptide can potentially self-assemble to well-defined nanostructures, rather than covering the metal surface more densely. In this case, the steric effects as a result of dense packing of conventional protective agents can be somewhat relieved. Given that the dispersion of Pd NPs increases with a greater exposed metal surface, their catalytic activity is improved accordingly.^37,38^ As such, the catalytic performances of peptide-templated Pd catalysts are often superior to Pd catalysts synthesized with conventional capping agents.

Beyond Pd catalysts, Knecht and his co-workers also studied the fabrication mechanisms of peptide-templated Au catalysts.^39,40^ Combined with theoretical and experimental results, they found that the strong binding between the peptide template and Au surface can be broadly defined as either entropically driven or enthalpically driven.^39^ The driving factor strongly depends on the number of anchor residues in the peptide sequence. When only a few anchors are spatially clustered at either end of the peptide sequence, entropically-driven binding occurs. As the other end of peptide remained unbound, this binding type allows the large entropic contribution to dominate the binding thermodynamics. On the contrary, if more anchor residues are spaced evenly along the peptide chain, the binding will be enthalpically driven. Therefore, the peptides are not irreversibly bound on the Au surface, which makes these peptides more amenable to Au surface modifications.^39^ Additionally, they further demonstrated that the peptide can control all facets of Au nanoparticle fabrication through both reducing and capping effects. This means that the addition of reducing agents like NaBH_4_ can be eliminated.^40^ They employed peptide AuBP1 (WAGAKRLVLRRE) and found the N-terminal tryptophan residue is responsible for reducing Au^3+^ to Au nanoparticles, with the reducing strength controllable by localized residue context effects. On the other hand, based on the theoretical results, they proved that the reducing residue of the peptide sequences does not impact their template function to Au nanoparticles, which suggests that the peptides can be used for all steps in the catalyst fabrication process.^40^

Besides Knecht’s efforts to synthesize peptide-templated Au catalysts and to study the peptide effects, other researchers have also made important contributions to develop peptide-templated syntheses.^41–45^ Walsh and his co-workers predicted the adsorption free energy of peptide AuBP1 at the aqueous Au (111) and Au (100) interfaces by employing an *in situ* combination of solute tempering replica exchange with metadynamic simulations.^41^ From the theoretical results, they found that the adsorption of AuBP1 on the Au (111) surface is stronger than that on the Au (100) surface.^41^ Fears *et al.* studied the interaction between Au surfaces and designed “GG-X-GG” “host–guest” sequences, where the central “X” residue was one of the 19 proteinogenic amino acids.^42^ It was found that the peptide with highest surface density is GG-C-GG, followed by peptide sequences with hydrophobic, charged, and polar central residues.^42^ Zhan *et al.* further developed a versatile technique to design peptide sequences for Au nanoparticle fabrication.^43^ Their novel technique relied on the modification of l-aspartic acid to combine one or two poly(ethylene glycol) (PEG) moieties and lipoic acid (LA) groups in the same sequence through a simple peptide organic process.^43^ With this designed peptide template, the obtained Au NPs exhibit great colloidal stability over a wide range of conditions. In an effort to arrange Au nanoparticles in a controlled fashion, Lamm *et al.* utilized laminated β-sheet fibrils as a template to generate parallel, linear gold nanoparticle arrays with a periodic lateral spacing. Their method took advantage of the complementary electrostatic interactions between negatively charged gold nanoparticles and the periodically arranged, positively charged lysine residues.^46^ These efforts make the peptide-templated synthesis a very effective technique for noble metal catalyst fabrication.

### 2.2 Peptide-templated noble metal nanofibers

As discussed in Knecht’s study, when the Pd/peptide ratio is high, linear/ribbon-like Pd nanostructures will be produced. When making such structures of catalyst, the peptide template and K_2_PdCl_4_ are added in the solution simultaneously, just like when making Pd nanoparticles. Besides this approach, another technique for producing noble metal catalysts with nanofiber structures is also developed by utilizing the self-assembly characteristic of peptides.^47–54^ Peptides or peptide conjugates with high propensity to form intermolecular hydrogen bonds are ideal molecular building units to construct one-dimensional supramolecular nanostructures. When employing this technique, the rationally designed peptide sequence first self-assembles into 1-D nanostructures in aqueous solution, followed by the addition of the noble metal precursor and reducing agent to fabricate one dimensional nanostructures on the peptide nanofiber templates, or by the direct addition of the synthesized nanoparticles having preferential binding to the surfaces of peptide-based nanostructures. Compared with procedures for producing peptide-templated nanoparticles, such synthesis processes are still under mild aqueous condition and simple to operate. However, the synthesis time would be longer since the peptides first need to self-assemble into nanofiber structures.

**Fig. 3:**
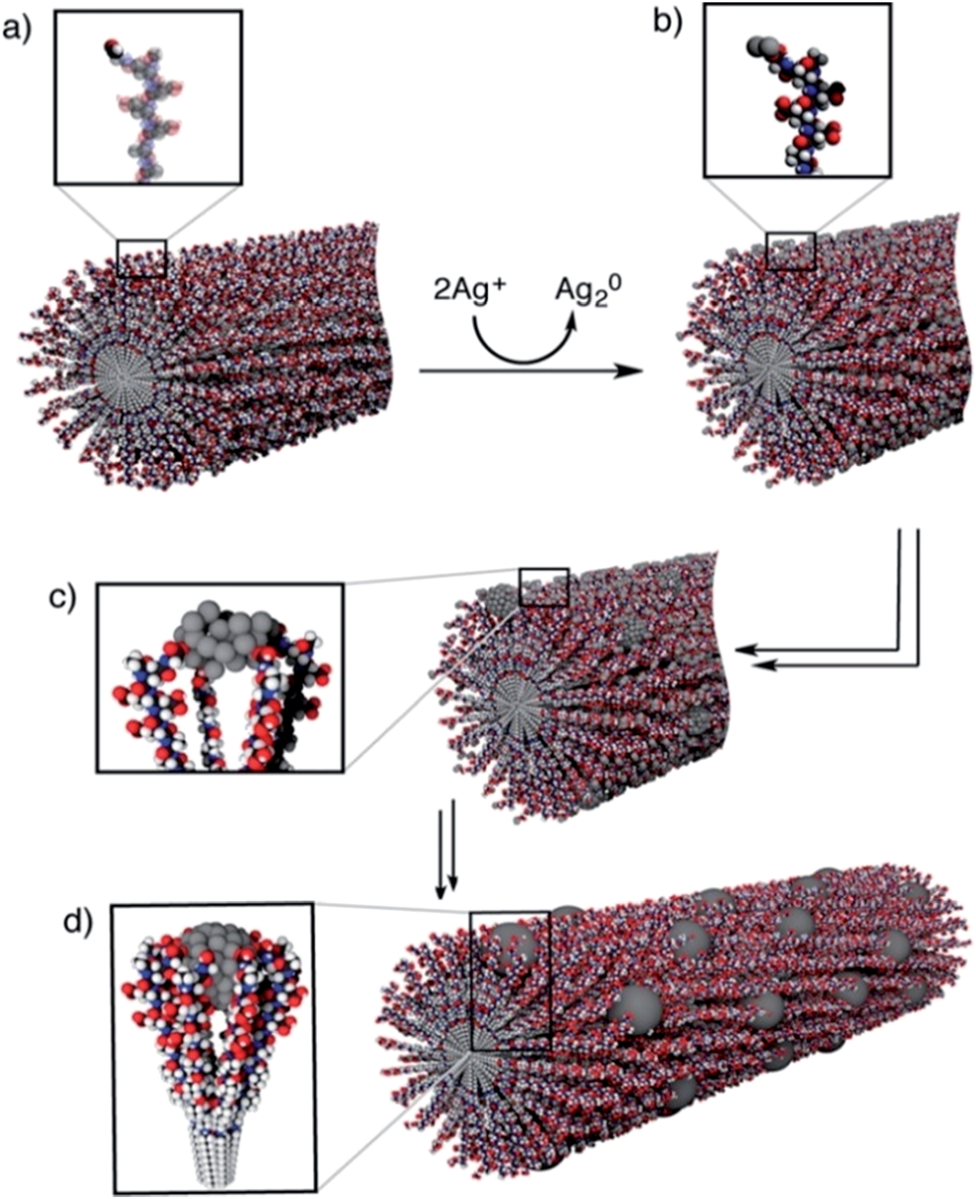
Formation mechanism of one-dimensional Ag nanostructures along a PA-1 nanofiber: (a) PA-1 self-assembles into a nanofiber with surface aldehyde functions; (b) formation of Ag_2_ clusters over the PA-1 nanofiber after the addition of Tollens’ reagent; (c, d) aggregation of silver clusters into larger nanoparticles and formation of a one dimensional structure, stabilized by the Glu residues. This figure is reproduced with permission from ref. 49, copyright American Chemistry Society.

The mechanism of peptide-nanofiber-templated synthesis has been discussed by Pazos *et al.*, who prepared ordered arrays of Ag nanostructures.^49^ As shown in Fig. 3, the whole synthesis process includes four steps. Firstly, the peptide PA-1 self-assembles into a nanofiber structure. After that, the Ag ions are reduced to clusters over the PA-1 nanofiber by Tollens’ reagent. Then, the Ag clusters aggregate into bigger nanoparticles. Lastly, Ag NPs grow continuously to form the ordered array nanostructures. According to UV-Vis and TEM results, they found that the growth and distribution of the obtained Ag NPs were directly controlled by the PA-1 nanofiber template due to the interactions between the Ag surface and carboxylate groups in Glu residues, as well as electrostatic repulsion and cohesive forces among PA-1 molecules.^49^ By using a peptide Ac-KFFAAK-Am nanofiber template and a multistep seed-mediated procedure, Guler and his co-workers prepared one dimensional resistive switching Au nanostructures.^50^ They found that the structure of the prepared Au catalyst changes from smooth nanowires, noodle-like one dimensional nanostructures to uniform aggregates of spherical nanoparticles as the Au/peptide and reductant/peptide ratio increase.^50^ In addition, Isozaki *et al.* ^51^ designed a novel Pt-complex-bound amino acid by combining a cyclometalated Pt complex with the side-chain residue of glutamic acid. After self-assembling into peptide nanofibers with such designed amino acids, the well-regulated Pt arrays are subsequently formed.^51^ Furthermore, Tomizaki *et al.* produced micrometre level ultrathin Au nanoribbons without any external reductants, while the Au nanoribbons are only 50–100 nm wide and 2.5 nm high.^52^ In order to study the formation mechanism of the Au nanoribbons, they designed and employed another four similar peptide sequences by changing one or two residues. Based on the TEM and AFM results, they demonstrated that the peptide can self-assemble into disk-like structures within the interior cavity of the network architectures. Then, the Au^3+^ ions will be reduced by electrons transferred from the naphthalene of peptide templates and the ultrathin Au nanoribbons are formed along the network architecture templates under ambient conditions.^52^

Modifications to the peptide template can also influence the characteristics of the resulting noble metal nanostructure. Merg and his co-workers showed that the number of gold-binding peptide heads (AYSSGAPPMPPF) and the length of conjugated aliphatic tails impact the characteristics of the formed Au double-helical nanofibers, such as pitch and interparticle distance.^53^ This peptide conjugate template gives rise to the notion of conjugating different moieties to peptides to produce a desired nanostructure.^53^ By combining peptide self-assembly characteristics and its specific control function, these reports have presented a new direction in the synthesis and application of peptide-templated noble metal catalysts.

### 2.3 Peptide–metal nanofilms

According to the summary above, it has been shown that researchers have synthesized noble metal catalysts through utilization of the self-assembly characteristics of peptides. Beyond 1-D structures, noble metal catalysts with 2-D structures can also be obtained by employing a peptide template whose nanofibers can undergo further associations to form nanofilms.^55,56^ One such example is provided by Jelinek and his co-workers, who synthesized transparent and conductive Au nanofiber films on a peptide monolayer.^56^ After peptide P_FK_-5 ((Phe-Lys)_5_-Pro) self-assembled into a nanofilm, a TEM grid was used to load the peptide film and then floated over an aqueous solution of Au(SCN)_4_^1–^. The negatively charged Au complexes bind to the peptide film and then reduce into metallic Au. After that, the nanofiber networks of Au are fabricated.^56^ According to such study, peptide-templated synthesis is an effective approach to fabricate noble metal catalysts with 2-D structures. However, due to the requirements of peptide self-assembly, this approach usually needs a longer operation time, which severely limits its development. By using an organic substrate to decrease the time cost, the catalytic performance of prepared catalysts will be influenced. Therefore, finding another novel method to reduce the time of the synthesis process is imperative.

Recently, a room temperature electron reduction^57–60^ with argon glow discharge as a cheap electron source has been developed to synthesize peptide-specifically-controlled noble metal nanoparticle composite films.^61,62^ Glow discharge is well known as a conventional cold plasma phenomenon with highly energetic electrons, with which the reduced metal NP surfaces feature mainly a (111) facet.^59–62^ This method needs no chemical reducing agents, protective agents or dispersing agents. In addition, the room temperature operation leads to the formation of metal NPs with small particle size and the addition of biological materials.^61,62^ With this method, Pan *et al.* reported the fast and simple fabrication of a self-assembled peptide nanofilm.^63^ In their study, an aqueous solution of the peptide KLVFFAE was dissolved in distilled water, exposed to argon glow discharge for 10 min, and then incubated at 37 °C. For samples treated by electron reduction, fibrils with diameters ranging from 40 to 100 nm and nanofilms were presented after incubating for 24 h and 120 h, respectively. On the contrary, for samples without electron reduction treatment, only countless peptide aggregations and fibrils with 20–80 nm diameter could be found after incubating for 24 h and 120 h, respectively. The evidently different results indicate that the electron reduction treatment can effectively reduce the time cost of the peptide assembly process. The reason behind these differences is due to the hydrated electrons formed during the treatment, with the peptide fibrils preferring to organize through C-to-C-terminal and N–(hydrated electron)–N-terminal interactions, instead of through C-to-N or N-to-N interactions, and therefore the peptide assembly rate increases significantly. When changing the sequence of the peptide to KLVFF and FKFEFKFE, the same phenomenon was also found, indicating that the electron reduction treatment can reduce the time cost of the self-assembly process of more peptide sequences.^63^

**Fig. 4:**
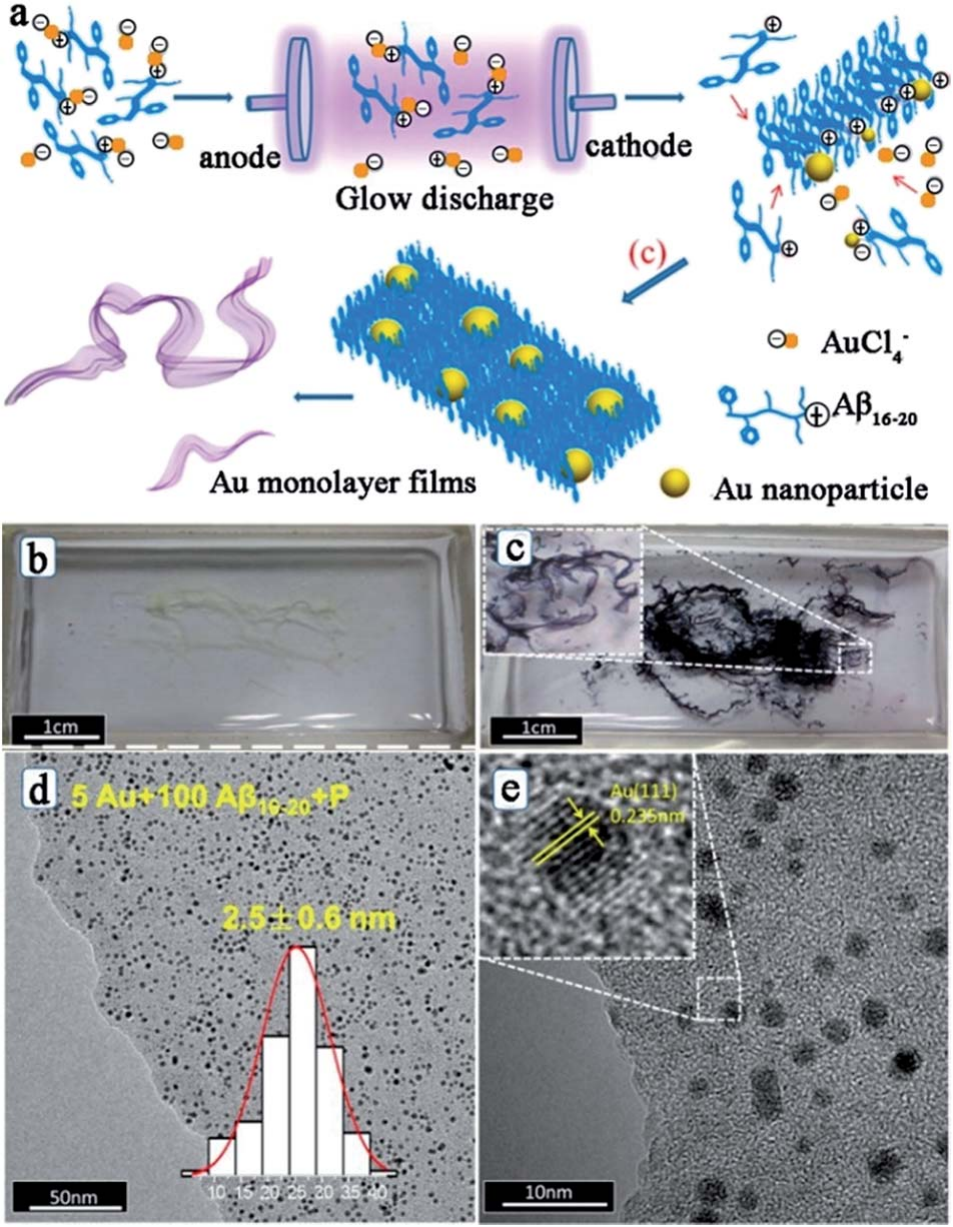
(a) Process of fabrication of a peptide–Au monolayer film: peptide templates interact with Au ions through the positive charge on the K residue firstly; next, Au ions are reduced to Au nanoparticles by electrons, while the peptide templates self-assemble into nanofibers; Au NPs and peptide nanofibers then assemble into well distributed films; (b) photograph of Aβ_16–20_ films and (c) Au/Aβ_16–20_ films in a quartz boat after the electron reduction; (d, e) typical TEM images of Au/Aβ_16–20_ films prepared by using 100 μM Aβ_16–20_ and 5 μM HAuCl_4_ hybrid aqueous solutions. The inset in (d) depicts the corresponding size distribution of Au NPs, and the inset in (e) shows the high resolution TEM image of the Au NP. This figure is reproduced with permission from ref. 62, copyright American Chemistry Society.

After studying the influences of the hydrated electron on the peptide assembly, Liu and his co-workers also reported the simultaneous synthesis of noble metal nanoparticles and peptide self-assembly structures by using the electron reduction method.^61,62,64^ Interestingly, after the electron reduction, the metal–peptide nanofilm is directly obtained, with the metal nanoparticles well-dispersed throughout the film. Fig. 4 shows the fabrication process, photographs, and morphologies of Au–peptide nanofilm.^62^ From Fig. 4, it can be observed that the formation process of the metal–peptide nanofilm is different from that of the chemical reduction approach. For chemical reduction fabrication, the peptide first self-assembles into a nanofilm and then the metal ions adsorbed on the peptide film are reduced to metallic nanoparticles to form a 2-D structure. For the electron reduction approach, the metal ions are reduced simultaneously during the peptide self-assembly process, so that the metal nanoparticles can disperse well on the peptide film and the time cost of the fabrication process is significantly reduced.^62^ Therefore, the electron reduction technique has opened a new route for peptide-templated noble metal catalyst fabrication.

## 3 Applications of the peptide-templated noble metal catalysts

Since the industrial application of Pt catalysts in the contact process for sulfuric acid production in 1875, noble metal catalysts have attracted great attention owing to their numerous applications including organic reactions, photocatalysis, electrocatalysis, and so on. The noble metal catalysts gain their excellent functionality from their inherent properties, which are typically a function of size, shape, facet, structure, and composition. As described above, peptide templates have an amazing ability to control nanoparticle size and dispersion, regulate nanoparticle facet, shape, and morphology, adjust surface chemistry, and establish the dimensionality of catalyst structures.^65,66^ This ability is well suited for noble metal catalysts with significantly enhanced catalytic activity and stability, by simply changing the peptide sequences and structures under ambient conditions.

### 3.1 Peptide-templated Pd catalysts

Owing to the high activity and selectivity, Pd catalysts have been employed for a variety of reactions including C–C couplings, such as Stille, Suzuki, and Heck couplings, as well as 4-nitrophenol reduction and olefin hydrogenation reactions.^67–70^ Traditionally, in order to enhance solubility and prevent bulk aggregation, homogeneous Pd catalysts for these reactions are usually in the form of complexes with phosphine ligands or other protective agents.^69^ However, the obtained Pd NPs are generally covered by a dense monolayer of protective agents, which may prevent the interaction of the reagents with the Pd surface and the Pd leaching process during oxidative addition, and this consequently leads to low catalytic activity.^71^ To overcome this issue, peptide templates have been applied for the syntheses of Pd catalysts with outstanding catalytic properties.

**Scheme 2:**
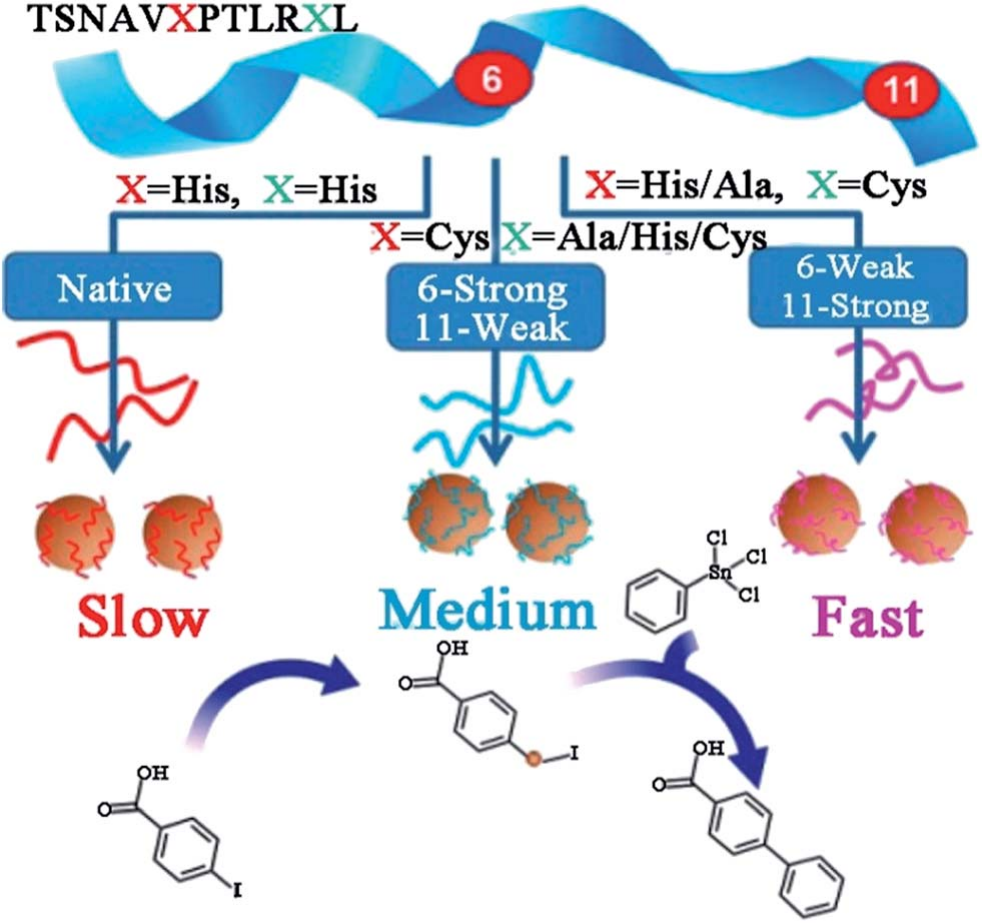
Effects of peptide sequence on the catalytic activity of peptide-templated Pd catalysts. This scheme is reproduced with permission from ref. 76, copyright American Chemistry Society.

Recently, Knecht, Naik and their co-workers have studied the synthesis and application of peptide-templated Pd catalysts comprehensively and thoroughly.^72–79^ They successfully synthesized a spherical Pd catalyst with the peptide Pd4 (TSNAVHPTLRHL) as the template.^72,73^ With a very low Pd concentration and reaction temperature, the prepared Pd catalyst showed high activity in Stille coupling reactions, especially for iodo-based reagents.^73^ Besides the Pd4 peptide template, they produced a series of equally sized spherical Pd catalysts with mutant peptides (A6, C6, A6C11, *etc.*) by replacing the histidine residues at positions 6 and 11 of the Pd4 peptide.^37,74–77^ Due to individual amino acid affinities and computationally predicted motifs of various peptide sequences, the surface coverage of Pd NPs, peptide–metal interactions, and ultimately the catalytic activity of prepared Pd catalysts differ significantly (Table 1).^37,76^ Notably, the mutant peptide with a cysteine substitution at position 11 (C11 peptide) generates the most active Pd catalyst and the highest turnover frequency for the Stille coupling reaction. For a traditional Pd catalyst, the coupling activity is usually diminished due to the coverage of protective agents. However, for a C11-templated Pd catalyst, in which the peptide strongly binds to the Pd nanoparticle surface through the cysteine thiol group, the remaining residues of the peptide are then arranged to provide optimal surface binding to minimize the surface energy.^76^ This binding mechanism will prevent additional surface coverage of the template and allow reagent access to the catalytic surface, and thus the Pd leaching process during oxidative addition is improved and the catalytic activity is enhanced (Scheme 2).^76^ This speculation had been corroborated by simulation, EXAFS and SAXS analyses.^37,77^

**Table 1:** Peptide sequence, Pd catalyst size, catalytic properties, and computed adsorption energies

Peptide	Sequence	Size^*a*^ (nm)	TOF^*b*^ (Stille coupling)	TOF^*b*^ (olefin hydrogenation)	Adsorption energy^*c*^ (kcal mol^–1^)
Pd4	TSNAVHPTLRHL	2.1 ± 0.4	2200 ± 100	5000 ± 200	–34.7 ± 1.1
A6	TSNAVAPTLRHL	2.2 ± 0.7	5200 ± 400	6100 ± 200	–26.8 ± 1.0
A11	TSNAVHPTLRAL	2.6 ± 0.4	1300 ± 10	2900 ± 700	–14.5 ± 1.0
A6,11	TSNAVAPTLRAL	2.8 ± 0.7	360 ± 20	2600 ± 300	–28.2 ± 1.0
C6	TSNAVCPTLRHL	2.2 ± 0.3	3960 ± 30	1300 ± 200	–23.0 ± 1.0
C11	TSNAVHPTLRCL	2.4 ± 0.4	6140 ± 60	800 ± 500	–24.0 ± 1.0
C6,11	TSNAVCPTLRCL	2.3 ± 0.4	4000 ± 300	0	–22.5 ± 1.0
C6A11	TSNAVCPTLRAL	2.4 ± 0.4	4100 ± 70	500 ± 200	–35.0 ± 1.0
A6C11	TSNAVAPTLRCL	2.4 ± 0.4	6100 ± 300	900 ± 100	–20.6 ± 1.0

^*a*^Average diameters from HRTEM.

^*b*^Moles product (mol_Pd_ × h)^–1^, 0.05 mol% Pd.

^*c*^The reported values are the computed adsorption energies per peptide molecule. This table is reproduced with permission from ref. 37, copyright American Chemistry Society.

**Fig. 5:**
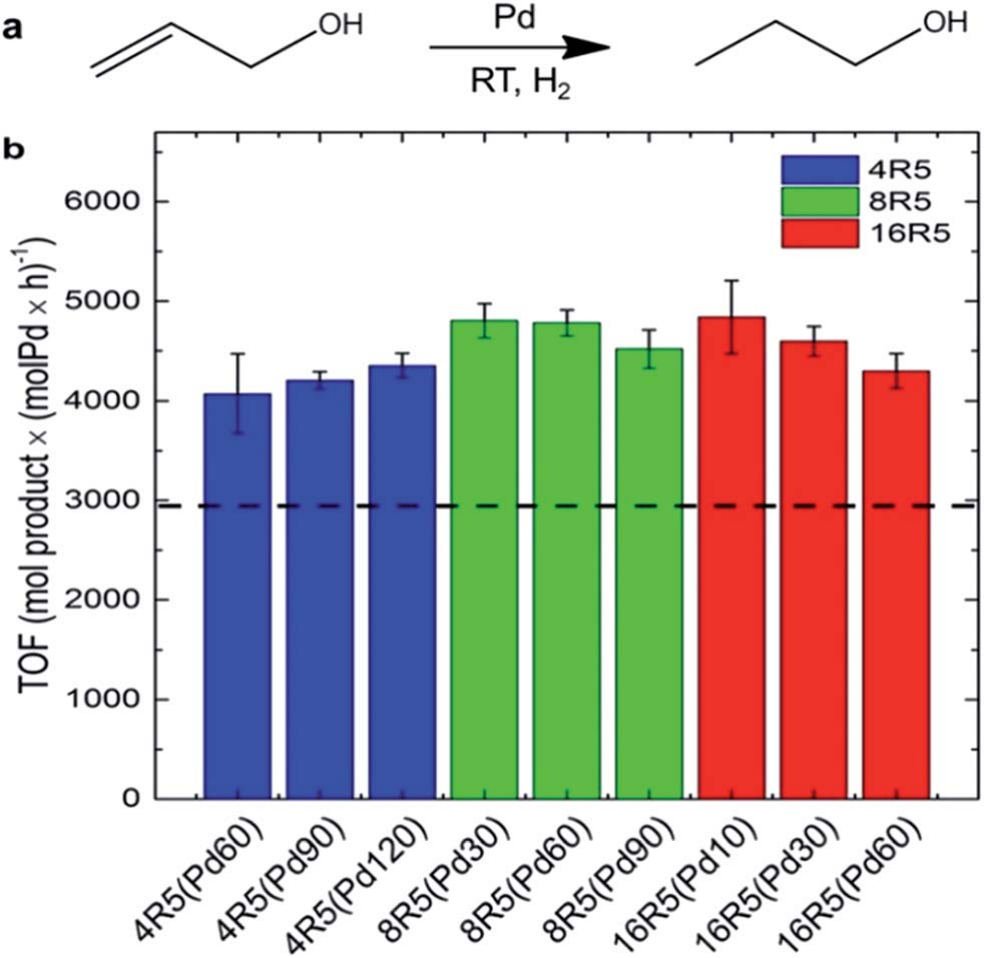
Catalytic analysis of the R5/dendrimer-templated Pd catalysts: (a) the reaction scheme and (b) TOF values for the hydrogenation of allyl alcohol. The black dashed line represents the average TOF value for Pd catalysts templated with only R5 peptide at ratios of 60–120 incremented by 10. This figure is reprinted with permission from ref. 79, copyright American Chemistry Society.

Beyond Pd4 and its mutant peptides, Knecht and Naik had also reported the fabrication of non-spherical Pd catalysts by using the peptide R5 (SSKKSGSYSGSKGSKRRIL) as the template.^78,79^ They found that by varying Pd/peptide ratios from 60 to 120, the shapes of Pd catalysts changed from spherical (Pd/peptide ratios = 60–80), linear/ribbon-like (90–110) to NPN (110–120) structure, but the size of these different Pd nanostructures was still constrictively controlled.^38,78^ Owing to the unique ability to load selective amounts of Pd materials into the scaffolds, both the metallic surface area and the penetration depth of reagents to the Pd surface can be flexibly altered. Therefore, these R5-templated Pd catalysts were proven to be highly active in the Stille coupling reaction, the 4-nitrophenol reduction reaction, and the hydrogenation of allyl alcohol, just like the Pd4-templated Pd catalysts.^38,78^ On the other hand, the surface areas and the penetration depth for oxidative addition differ for Pd NPs of different shapes, thus leading to changes in their catalytic properties.^38^ For spherical Pd catalyst, there exist the highest Pd surface and the largest penetration depth as a result of the high dispersity of Pd NPs. In stark contrast, for Pd NPNs, the exposed Pd surface area is the lowest and the penetration depth is the smallest because the highest Pd loading causes the reactive surface closer to the peptide/water interface. The catalytic performances of spherical Pd catalyst (452 mol product (mol_Pd_ × h)^–1^) and Pd NPNs (437 mol product (mol_Pd_ × h)^–1^) are therefore similar. However, for anisotropic Pd catalyst, the reactive surface is smaller than that of spherical Pd catalyst, while the penetration depth is larger compared with Pd NPNs. With these two negative structural characteristics, the anisotropic Pd catalyst reveals the lowest catalytic activity (334 mol product (mol_Pd_ × h)^–1^) among these three types of Pd catalysts.^38^ It is therefore clear that the shape of Pd catalysts has important influence on their catalytic performance. Furthermore, by attaching R5 peptide to the surface of small and water-soluble dendrimers to form the modified peptide, the prepared Pd catalysts showed much higher catalytic activity than R5-templated Pd catalysts (Fig. 5).^79^

Besides studies by Knecht and Naik, the applications of Pd catalysts synthesized with other peptide sequences are also reported. Guler and his co-workers fabricated Pd(0) hybrid nanocatalysts (Pd@peptide) on the peptide nanofiber template (Lauryl-VVAGHH-Am). After catalysing the Suzuki coupling reaction effectively, the Pd@peptide catalyst could be easily separated and reused in successive reactions without significant structural integrity and loss in activity.^80^ Maity *et al.* produced Pd catalysts on the peptide nanofiber too, while the nanofiber was formed by the peptide bolaamphiphile 1 (HO-Y-W-Suc-W-Y-OH).^81^ The obtained catalyst enabled high catalytic activity in the reduction of nitrophenol isomers at room temperature. By conjugating a Pd4 peptide to an oligomeric peptide p53Tet (p53 tetramerization domain), Janairo *et al.* designed a new peptide sequence.^82^ With this novel template, they prepared branched, coral-like Pd catalysts, which showed enhanced activity in reduction of nitrophenol.^82^ According to the studies discussed above, Pd catalysts synthesized with various peptide sequences have revealed excellent catalytic behaviours in many organic reactions, which follow drastically different catalytic mechanisms. Furthermore, Chen *et al.* employed Pd4 series peptide-templated Pd catalysts for the oxygen reduction reaction (ORR) with enhanced electrocatalytic activity.^83^ The observed relationships between the electrocatalytic activity and the peptide sequences suggest that the residue-specific binding effects also have significant impact on the electron transfer properties of peptide-templated Pd catalysts.^83^ These results suggest that the peptide template synthesis is an effective approach to fabricate highly active Pd catalysts.

### 3.2 Peptide-templated Au catalysts

To date, peptide-templated Au nanomaterials have been widely employed in biological applications, such as protein activity inhibition,^84^ integrin detection^85^ and antibody sensors.^86,87^ Beyond these applications, peptide-templated Au nanomaterials have been proven to be good catalysts and have attracted great attention during the past years.^88–95^ It has been demonstrated that the catalytic activity of the Au catalyst is directly influenced by the localized surface plasmon resonance (LSPR) of Au NPs, which is sensitive to the size, shape, and aggregation state of the catalysts.^96^ Similarly to peptide-templated Pd catalysts, these inherent characteristics of Au catalysts can be specifically controlled by peptide templates, so that the LSPR of Au NPs can be tuned and consequently their catalytic activity can be improved.^87,88^

**Scheme 3:**
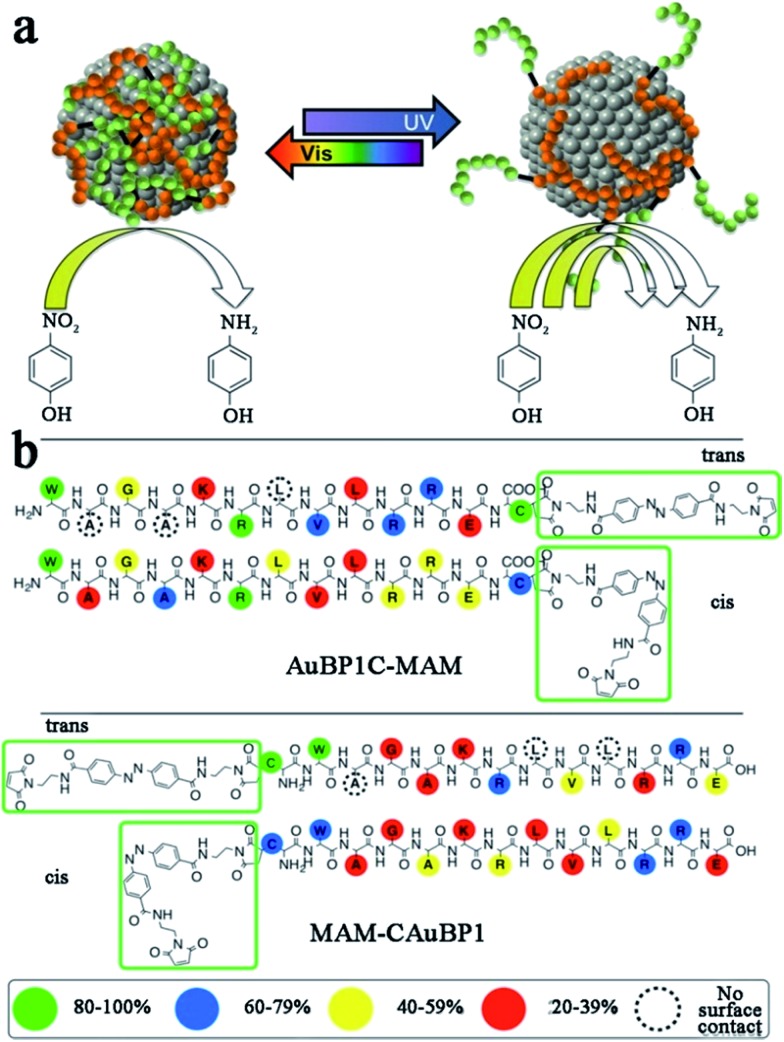
(a) Due to the photo-switching of the biomolecular structure, the metal surface exposure could be changed to control the catalytic activities; (b) contact score analysis for the hybrid biomolecules. The binding of the overall biomolecule to the Au surface varies based upon the position and isomerization state of the MAM unit. This scheme is reproduced with permission from ref. 92, copyright American Chemistry Society.

Based on studies of peptide-templated Pd catalysts, Knecht and his co-workers synthesized a series of Au catalysts with different peptide sequences.^88–92^ With an R5 peptide self-assembly scaffold, they first synthesized non-spherical Au nanoparticle networks (Au NPNs), which showed outstanding activity in 4-nitrophenol reduction reactions.^88^ Due to the strong interaction between the Au surface and peptide, the morphology of the R5-templated Au catalysts will not change with variations in the Au/peptide ratio.^88^ Besides the R5 template, they further tested the 4-nitrophenol reduction activity of Au catalysts templated with ten different peptide sequences.^89^ They found that the peptide–Au interface plays multiple roles in controlling the reactivity through steric and electronic effects. Among these, steric effects likely play a dominant role in the reactivity. For example, the peptide template with more strongly-binding residues tends to lay flat on the Au surface, so that the access of the bulky 4-nitrophenol reagent to the catalytic surface will be limited and consequently the reactivity will be lowered.^89^ This speculation of the relationship between the peptide sequence and catalytic activity of Au catalysts was later confirmed by molecular dynamic (MD) simulations.^90^ After studying the influence of peptide sequences on the activity of Au catalysts, the role of reducing agents was discussed.^91^ When using weak reductants like ascorbic acid, large and globular Au NPs with rough surfaces are fabricated. On the contrary, strong reductants like NaBH_4_ produce small, spherical, and smooth Au NPs, which show higher catalytic activity.^91^ Furthermore, Knecht *et al.* also tried to modify the peptide template to improve catalytic behaviours of Au catalysts.^92,93^ Different from the modification of Pd peptides with dendrimer addition, they attached two photo-switchable azobenzene maleimide groups (MAM) to the N- and C-termini of the parent AuBP1 sequence, to form MAM-CAuBP1 and AuBP1C-MAM sequences, respectively (Scheme 3). Due to the optical switching effects of MAM isomerization states, the adsorption motifs and coverage of modified peptide templates on the Au NPs surface will be changed when introducing different light. Then, the catalytic activity of prepared Au catalysts in the 4-nitrophenol reduction reaction can be directly controlled.^92^ This strategy opened a new pathway toward achieving remotely manipulated reactivity, where enhancements in such capabilities can be achieved through peptide sequence design.

Aside from Knecht’s studies, Merg *et al.* fabricated single-helical gold nanoparticle superstructures with high chiroptical activity.^94^ The peptide template they used was C18-(PEP_Au_^M-ox^)_2_ (PEP_Au_^M-ox^ = AYSSGAPPMoxPPF).^94^ Tang and his co-workers prepared electrocatalytic Au catalysts with an R5 peptide as the template.^95^ They found that the prepared Au catalyst with the Au/peptide ratio of 90 shows the best electrocatalytic activity.^95^ These results indicate that, beyond biological applications, the peptide-templated Au materials show good performances in catalysis as well.

### 3.3 Peptide-templated Pt catalysts

Nowadays, Pt catalysts are still the most widely used catalyst in photocatalysis and electrocatalysis, since Pt nanoparticles can expand the light absorption band into the visible light region^96^ and lower the electrochemical overpotentials by facilitating the charge transfer.^97^ However, due to the low efficiency in separation/transfer of the photoinduced electrons^61^ or the sluggish kinetics of the ORR at the cathode,^64^ it is still necessary to develop novel Pt catalysts to gain higher activity. Compared with traditional catalysts, peptide-templated Pt catalysts with specific characteristics have revealed evident catalytic improvements in photocatalysis and electrocatalysis, since the electron conductivity and Pt dispersion are both enhanced significantly with peptide assistance.^98–101^

**Fig. 6:**
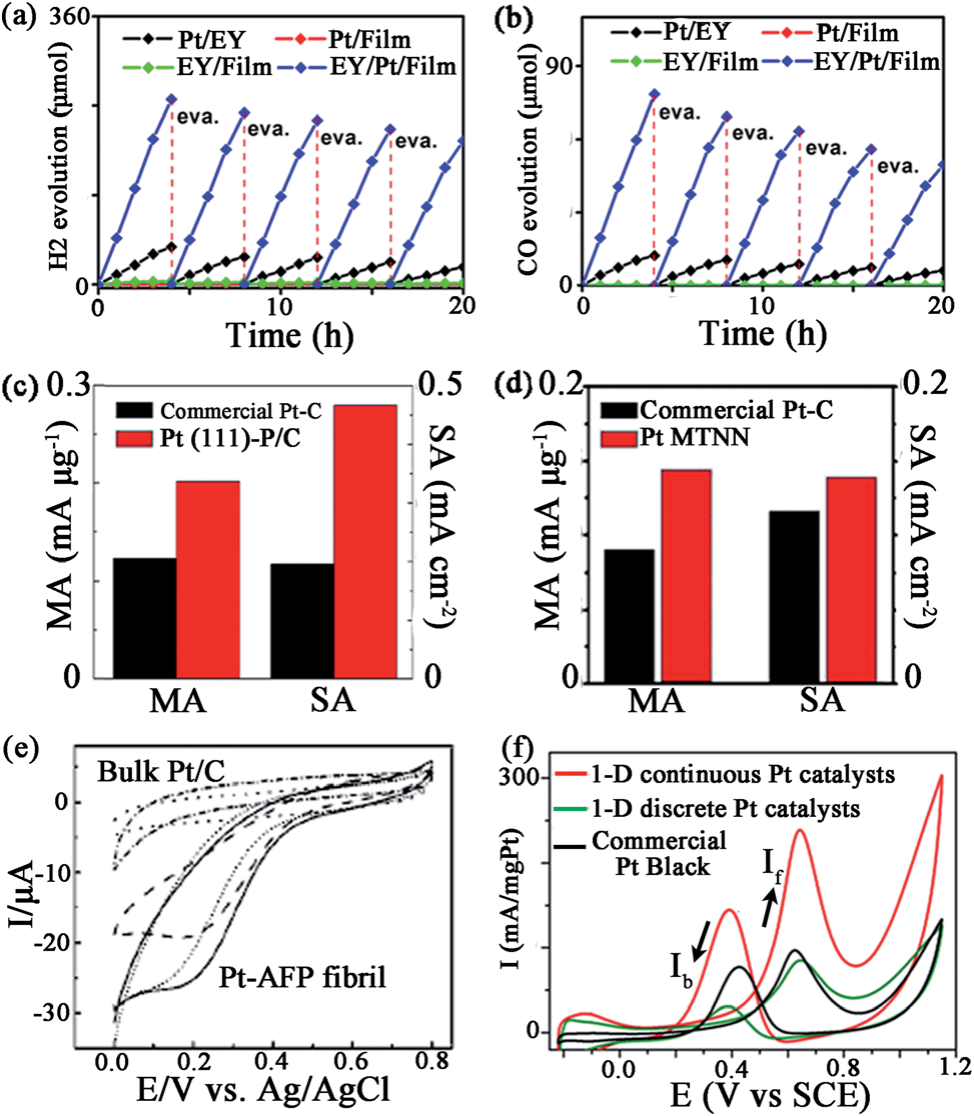
Some catalytic activities of peptide-templated Pt catalysts: (a) H_2_ evolution rate and (b) CO evolution rate of EY/Pt/film catalyst, ORR activities of (c) Pt (111)–P/C and (d) Pt MTNN catalysts, (e) CV curves of the dioxygen reduction of Pt–AFP fibril catalyst and (f) CV curves of the methanol oxidation of the 1-D Pt nanostructure catalysts. (a, b) is reproduced with permission from ref. 61, copyright American Chemistry Society; (c) is reproduced with permission from ref. 64 copyright Elsevier; (d) is reproduced with permission from ref. 99, copyright John Wiley and Sons; (e) is reproduced with permission from ref. 100, copyright Royal Society Chemistry; (f) is reproduced with permission from ref. 101, copyright PubMed Central.

Pan and his co-workers found that with a Pt–peptide nanofilm as the support, the EY (Eosin Y)/Pt/film catalyst is a promising catalyst for photocatalytic water splitting to H_2_ and photocatalytic CO_2_ reduction with water to CO, under irradiation of visible light.^61^ Interestingly, the peptide film acts not only as a capping agent to control the small size and high dispersion of Pt nanoparticles, but also as a support to load EY *via* gently stirring a solution containing EY and Pt/film. Compared with Pt/EY (11.8 μmol h^–1^) and EY/Pt/TiO_2_ (41.4 μmol h^–1^) catalysts, EY/Pt/film exhibits an enhanced H_2_ evolution rate of 62.1 μmol h^–1^ (Fig. 6a). The CO evolution rate on EY/Pt/film is 19.4 μmol h^–1^, which is 7 times higher than that on Pt/EY and 3.2 times higher than that on the EY/Pt/TiO_2_ catalyst (Fig. 6b). The outstanding activity of EY/Pt/film is originated from more photoinduced electrons participating in the reaction with H_2_O (or CO_2_ + H_2_O), since the transfer of photoinduced electrons from EY to the Pt nanoparticles is promoted due to the high conductivity of the peptide nanofilm.^61^

Furthermore, Wang *et al.* also proved that Pt–peptide nanofilm-based Pt catalysts (Pt (111)–P/C catalyst) exhibit excellent catalytic behaviours in ORR.^64^ The Pt (111)–P/C catalyst is simply prepared by loading a Pt–peptide nanofilm on a carbon black support, while the prepared catalyst shows surface sites featuring predominately (111) facets with particle sizes around 2 nm. The mass activity and specific activity of Pt (111)–P/C are 0.202 mA μg^–1^ and 0.467 mA cm^–2^, which are 1.6 and 2.4 times greater than those of the commercial Pt/C catalysts, respectively (Fig. 6c). The peptide plays a key role in the high ORR activity of the Pt (111)–P/C catalyst. With the specific controlling of the peptide, the oxygen binding on Pt NPs of the Pt (111)–P/C catalyst is much weaker than that of the commercial Pt/C catalyst. On the other hand, the hydrophilic property and the electron conductivity of the carbon support are also improved, owing to the assistance of peptide. That is why the Pt (111)–P/C catalyst reveals a higher ORR activity than that of commercial Pt/C catalysts.^64^

Besides a Pt–peptide nanofilm fabricated with peptide KLVFF, the peptide-templated Pt nanocrystal and nanowire are also proven to have outstanding performances in electrocatalysis. Huang and her co-workers fabricated Pt nanoparticles^98^ and an ultrathin Pt multiple-twinned nanowire network (Pt MTNN)^99^ with peptide BP7A (TLHVSSY-CONH_2_) as the template. With K_2_PtCl_6_ as the precursor, they found that when the peptide concentration increases from 22.5 to 250 μg mL^–1^, the shape of Pt catalysts changes from NPs with long pods to NPs with short pods and then to spherical NPs.^98^ Among these Pt catalysts, the Pt NPs with long pods show the largest electrochemical surface area (ECSA), while spherical Pt NPs show the smallest ECSA. The decrease of ECSA of different Pt catalysts might result from relatively more peptide molecules remaining on the Pt NPs with shorter pods or spherical Pt NPs since they are synthesized at higher peptide concentrations.^98^ On the other hand, by changing the Pt precursor, the shape of Pt catalyst changes to Pt MTNN.^99^ The Pt MTNN catalyst also shows significantly enhanced activity in both ORR and MOR (methanol oxidation reaction) due to more structural defects made by a large population of twin planes (Fig. 6d).^99^ In addition, Zhou *et al.* ^100^ directly immobilized the negatively charged Pt nanoparticles evenly onto the positively charged surface of AFP (aniline-GGAAKLVFF) self-assembled fibrils *via* an electrostatic interaction technique, such that the morphology and dispersion of Pt NPs are strictly controlled and the catalytic performance is improved consequently (Fig. 6e). By associating the two techniques above, Tao *et al.* ^101^ synthesized 1-D Pt nanostructures with the fibrils formed by peptide I_3_K (Ac-IIIK-CONH_2_) as the support and the peptide P7A (TLHVSSY) as the capping agent. As shown in Fig. 6f, the MOR activity and CO tolerance of as-prepared Pt catalyst are evidently increased. These studies suggest that the peptide template synthesis is an effective approach to prepare highly active and stable Pt catalysts.

### 3.4 Peptide-templated bimetallic catalysts

While noble metal catalysts show outstanding catalytic behaviours in many applications, bimetallic catalysts are considered to evidently enhance the functionality of the catalysts.^102–105^ This enhancement effect likely originates from intimate interactions between the two components, which may cause the geometric arrangement of the two metal components or electronic improvements to the catalysts.^106^ For peptide-templated monometallic catalysts, it has been demonstrated that the peptide can effectively control the structure and morphology properties and improve the electron conductivity of catalysts. Therefore, many attempts have been made to fabricate peptide-templated bimetallic catalysts in recent years.^107–110^

**Fig. 7:**
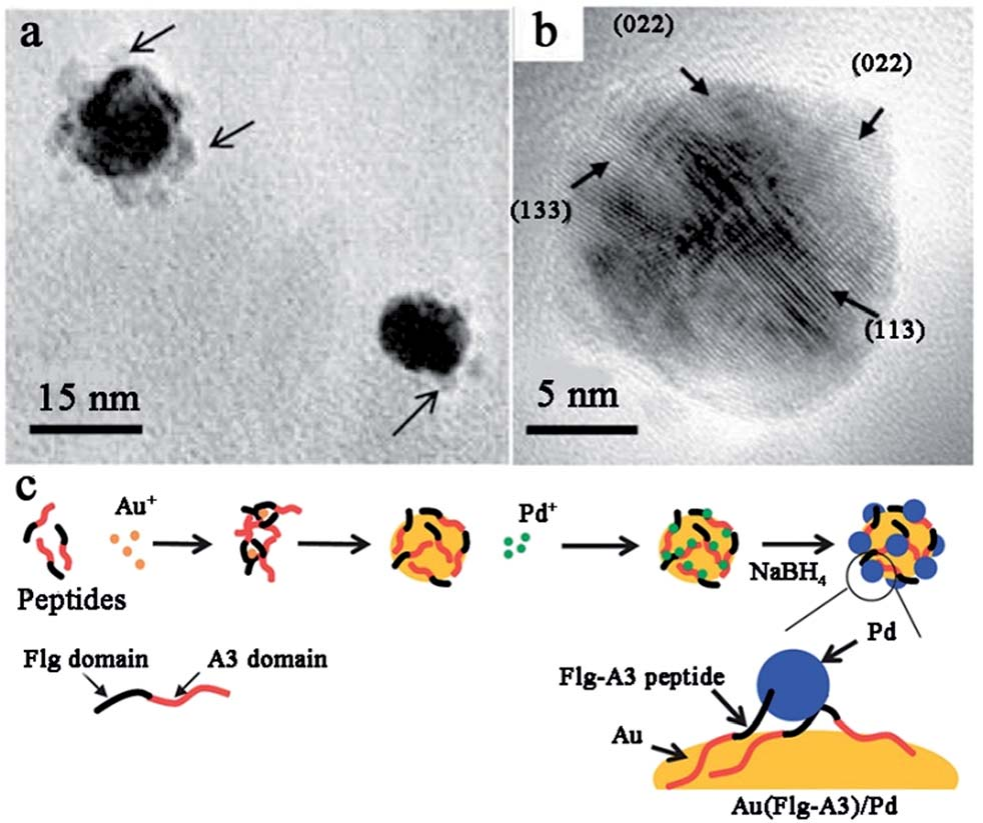
Peptide-templated synthesis of Pd–Au bimetallic catalysts. (a) The low magnification TEM image of Au–Pd bimetallic nanoparticles; (b) the HR-TEM image of the Au–Pd bimetallic NPs displaying the crystal lattices of Pd present along the Au nanoparticle surface; (c) the synthetic scheme of Pd–Au bimetallic catalysts *via* the use of a fusion peptide sequence. This figure is reproduced with permission from ref. 107, copyright John Wiley and Sons.

In order to prepare Pd–Au bimetallic catalysts using a peptide template, Naik and his co-workers employed Flg-A3 (DYKDDDDK-AYSSGAPPMPPF) multifunctional peptide, which is formed by the fusion of two peptide binding sequences.^107^ As shown in Fig. 7, when preparing the Pd–Au bimetallic catalyst, the A3 domain of the template will first bind to the surface of Au NPs and expose the Flg domain to the solution for Pd ion binding. Then, the Pd ion will be reduced by NaBH_4_ on the Au surface to form a Pd–Au bimetallic catalyst. Notably, the size of the prepared bimetallic catalyst is only 3 nm due to the control ability of the peptide template. This catalyst shows high activity in hydrogenation of 3-butenol to 1-butanol, while the turnover frequency (TOF) can reach up to 1016 mol product per hour (mol_Pd_ × h)^–1^.^107^ Besides the Flg-A3 peptide, AuBP1, H1, and Pd4 peptides were all employed by Bedford *et al.* ^108^ to study the effect of the peptide sequence on the structure, composition and electrochemical activity of methanol oxidation over Pd–Au bimetallic catalysts. By associating theoretical and experimental results, they found all obtained bimetallic nanoparticles are ∼2 nm in size and indicated a surface enrichment of Pd, which is attributed to strong Pd–peptide interactions dependent on peptide sequence. However, the Pd surface content will increase from 66.4% to 73.8% when using the Pd4 peptide instead of AuBP1, since the interaction between the Pd4 peptide and Pd surface is stronger than that of the AuBP1 peptide. Furthermore, the Pd4 peptide-templated Pd–Au catalyst shows improved electrocatalytic activity, as more Pd atoms exist on the surface.^108^

Besides Pd–Au bimetallic catalysts, Slocik *et al.* ^109^ reported the fabrication of an integrated CdS–Pt nanoparticle system. The catalyst comprises CdS quantum dots that are surface decorated by catalytic Pt nanoparticles. The structure is achieved by using the cysteine-modified Flg-A3 peptide, the same as the forming mechanism of the Pd–Au bimetallic catalyst described above. With assistance of the peptide, the electron transfer of the catalyst is enhanced, so that the catalyst reveals outstanding catalytic behaviours in nitrate reduction using photochemical-based approaches.^109^ Furthermore, by using the novel peptide template BP-PEP_Co_ (C_12_H_9_CO-HYPTLPLGSSTY), Rosi and his co-workers prepared CoPt nanoparticles with hollow spherical magnetic superstructures, which can be used as catalysts for the oxidation of methanol.^110^ Different from other peptide-templated catalysts, the size of CoPt hollow superstructure reaches as large as 53.9 ± 7.9 nm. However, when using peptide PEP_Co_ as the template, only random aggregates composed of individual nanoparticles exist and the size of the nanoparticles decreases significantly to only 2.61 ± 0.43 nm.^110^ This interesting result indicates that the size and structure of catalysts can be effectively controlled by different peptide templates, providing a versatile route for achieving high activity catalysts.

## 4 Conclusions and outlook

From the practical perspective of nanoscale science and technology, to synthesize noble metal catalysts with desired properties using the bottom-up approach, as nature does, is one of the ultimate goals. Recently, increasing studies on the synthesis and application of peptide-templated noble metal catalysts have proven that peptides can serve as excellent templates for specific control of the size, shape, facet, structure, orientation, and composition of prepared catalysts. As displayed in our discussed examples, the synthesis conditions of the peptide-templated approach are much milder than those of traditional fabrication processes. As a result of the different binding affinities and interactions between the peptide and the specific metallic surface, noble metal catalysts with various morphologies and specific characteristics can be produced by simply varying the peptide sequences, metal/peptide ratios, and metal precursors. Therefore the exposed metal surface area and the penetration depth of noble metal catalysts can be both tuned. By a series of theoretical and experimental studies, the interactions between the peptide template and metal surface have been investigated, as well as the adsorption motifs and energies of peptides on the metal surfaces. Different from conventional protective agents, the peptide template adsorbs on the surface of noble metal NPs through the strong interactions between several residues and metallic sites, while the remaining residues of the peptide are then arranged to provide optimal surface binding to minimize the surface energy. Therefore, the steric effects of conventional protective agents can be overcome. Furthermore, combined with the peptides’ self-assembly properties, catalysts with 1-D and 2-D structures can also be prepared and modified. Additionally, as the electron conductivity, metal dispersion, and reactive site exposure are all improved by the peptide template, the prepared noble metal catalysts show significantly enhanced catalytic performances in organic reactions, photocatalysis, and electrocatalysis. Besides that, the relationships between catalytic properties and the specific facet of noble metal catalysts can be studied directly as the nanoscale single crystals can be fabricated easily with peptide templates. What’s more, with the assistance of peptide templates, a series of noble metal catalysts with different particle sizes can be synthesized. By this method, the influences of particle size on all characteristics of noble metal catalysts, like localized surface plasmon resonance, can be investigated easily and systematically. Finally, for producing bimetallic catalysts, both the structure and composition can be controlled as desired under the assistance of a peptide template, while the fabrication process is simple and fast with low energy cost. These advantages suggest that the peptide is an effective and novel template for noble metal catalysts syntheses and applications.

On the other hand, there are still some issues that limit further application of peptide-templated syntheses. Recent efforts have greatly enhanced our knowledge about the interactions between the peptide and metallic surface *via* modified theoretical calculations. However, the library of peptide functions or molecular binding motifs on different metal surfaces needs further enrichment. The limitations on the minimal peptide length and sequence composition should be investigated, as this could significantly simplify the synthesis process for noble metal catalysts and scale-up requirements. Moreover, the development of a universal peptide/polypeptide/protein structure that allows for the formation of nanostructures for a wide variety of metals and other inorganic materials would be beneficial to simplify syntheses. The functionality of peptide-templated noble metal catalysts could be further expanded by association with proteolytic enzyme activity, which may serve to improve catalytic activity after cleavage from peptides. It is also known that many helical peptides have piezoelectric and other interesting electronic properties. Combining these characteristics with the catalytic activities of noble metal nanoparticles may open up more opportunities for exploration with possible new research directions.

Moreover, though simulation results and formation mechanisms have been indirectly proven by TEM, AFM, EXAFS, and SAXS measurements, new techniques, including spectroscopy and microscopy (containing high-resolution and staining methods), that allow for the characterization at the molecular level are necessary in order to completely and directly define the peptide–metal interface. Furthermore, besides Pd, Au, and Pt, the peptide-templated syntheses of other noble metals with high catalytic activity (like Rh, Ru and Ir) need to be further developed. Finally, for peptide-templated catalysts with 1-D, 2-D and 3-D structures fabricated with various novel approaches, the formation mechanisms and the structure–property relations still need to be addressed. Further theoretical calculations and experimental studies are immediately needed.
